# Dual-release hydrocortisone vs conventional glucocorticoids in adrenal insufficiency

**DOI:** 10.1530/EC-19-0176

**Published:** 2019-06-04

**Authors:** V Guarnotta, C Di Stefano, A Santoro, A Ciresi, A Coppola, C Giordano

**Affiliations:** 1 Dipartimento di Promozione della Salute, Materno – Infantile, Medicina Interna e Specialistica di Eccellenza ‘G. D’Alessandro’ (PROMISE), Sezione di Malattie Endocrine, del Ricambio e della Nutrizione, Università di Palermo, Palermo, Italy

**Keywords:** dual-release hydrocortisone, cardiovascular risk, diabetes mellitus, adrenal insufficiency, conventional glucocorticoids

## Abstract

**Background:**

Dual-release hydrocortisone (DR-HC) improves metabolism in patients with adrenal insufficiency. The aims of this study were to compare the cardiovascular and metabolic effects of conventional glucocorticoids (GCs) vs. DR-HC and of high vs. low doses of GCs, after 48 months of observation.

**Methods:**

We selected 27 patients on hydrocortisone (mean dose 17.5 ± 4.2 mg/day) and 20 patients on cortisone acetate (mean dose 37.5 ± 12.1 mg/day) who maintained this treatment (group A) and 53 patients switched to DR-HC (mean dose 22 ± 4.8 mg/day) (group B). At baseline and after 48 months, clinical and metabolic parameters and Framingham Risk Score (FRS) were obtained.

**Results:**

After 48 months, patients in group A had a significant increase from baseline in BMI (*P* < 0.001), waist circumference (*P* = 0.001), systolic blood pressure (*P* = 0.001), LDL cholesterol (*P* = 0.018), HbA1c (*P* = 0.020) and FRS (*P* = 0.002). By contrast, patients in group B had a significant decrease in BMI (*P* = 0.002), waist circumference (*P* = 0.015), diastolic blood pressure (*P* = 0.031), total (*P* = 0.006) and LDL cholesterol (*P* = 0.005), HbA1c (*P* < 0.001) and FRS (*P* = 0.015) compared to baseline. No significant differences between high and low doses of both conventional GCs and DR-HC were observed.

**Conclusions:**

DR-HC is associated with an improvement of metabolic parameters and cardiovascular risk compared to conventional GCs, which are associated with a worsening of these parameters, regardless of the dose used.

## Introduction

Adrenal insufficiency (AI) is characterized by high morbidity and mortality, likely due to inappropriate glucocorticoid (GC) treatment and no physiological daily exposure. Indeed, conventional GC treatment, with hydrocortisone (HC) or cortisone acetate, requires two-three daily doses to maintain adequate plasma cortisol levels, with the highest dose administered in the morning and a lower dose in the afternoon or, if required, in the evening (1), exposing patients to supraphysiological levels of cortisol. Overexposure to GCs has been demonstrated to increase metabolic dysfunction and cardio metabolic risk and cause a sleep pattern disturbance, resulting in impaired quality of life and enhancing mortality and morbidity (2, 3).

Once-daily dual-release hydrocortisone (DR-HC) has been demonstrated to provide a cortisol exposure-time profile close to the physiological one. It is characterized by an immediate-release fraction of HC in the outer layer of the tablet and an extended-release fraction in the core, able to provide an adequate concentration of cortisol within 50 min of administration, half-cortisol plasma concentration for 6 h thereafter and a minimal cortisol level 18–24 hours after intake (4). This characteristic formulation has been shown to improve anthropometric and metabolic parameters and quality of life, appearing to be safe in the long term (5, 6, 7, 8, 9, 10).

The primary objective of the current study was to compare the effects of DR-HC and conventional GCs in patients with AI, on anthropometric parameters, glucose and lipid metabolism, and cardiovascular risk estimated by the FRS for non-diabetic patients (11) and the United Kingdom Prospective Diabetes Study (UKPDS) risk engine for diabetic patients (12), after 48 months of treatment. Secondary objective was evaluating the differences in anthropometric and metabolic parameters between patients treated with high and low doses of both conventional GCs and DR-HC at 48 months.

## Materials and methods

### Study participants

**Figure 1:**
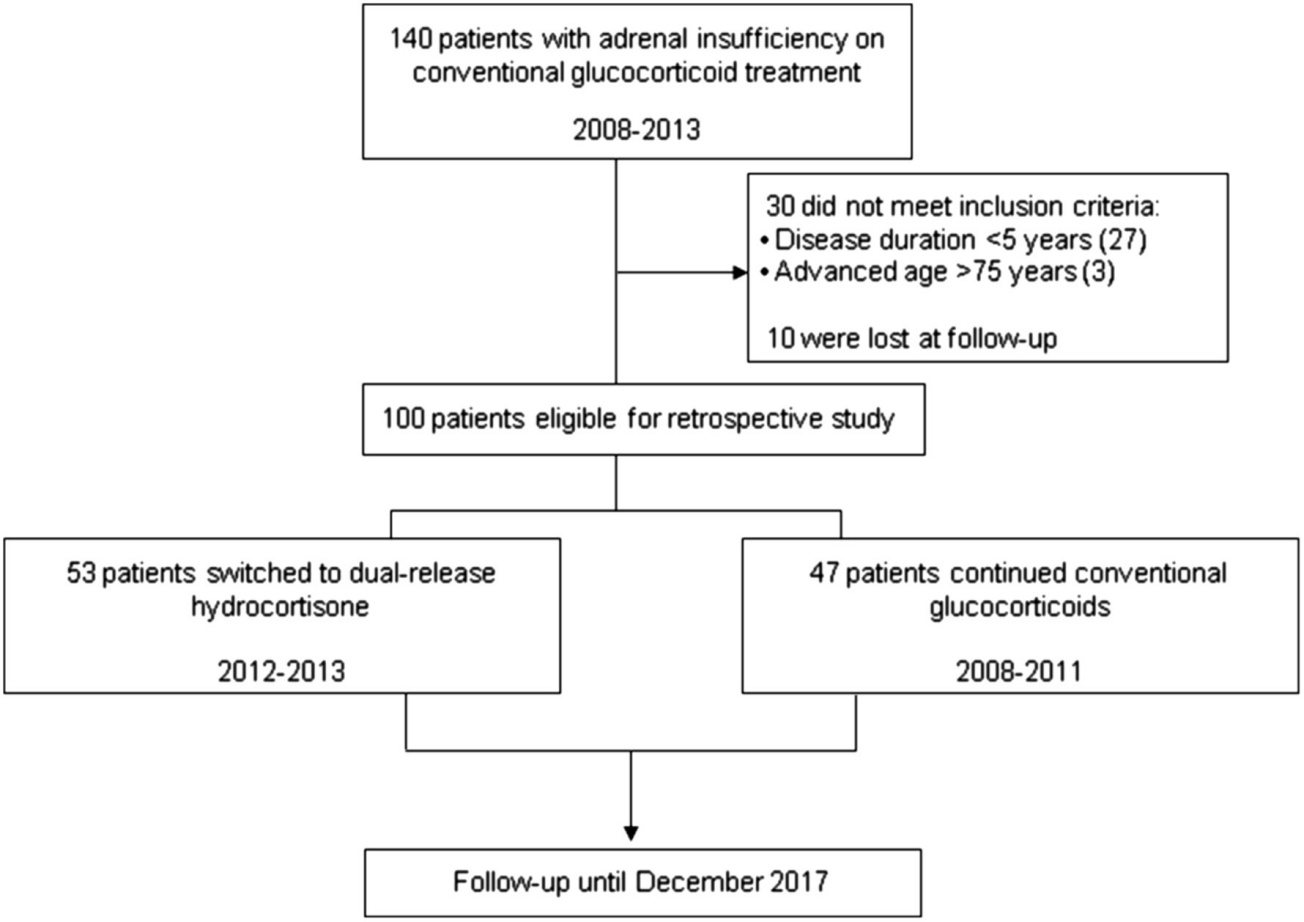
Flow chart of patient enrolment during the 48-month period of observation.

We evaluated data from 100 consecutive patients, 44 with PAI and 56 with SAI due to hypopituitarism, out of a total of 140 patients who were on conventional GC treatment. Patients were consecutively referred to the Division of Endocrinology of Palermo University from January 2008 to December 2013. Specifically, we carefully selected data of 47 patients who were on conventional GC treatment (20 on cortisone acetate and 27 on HC), administered twice or three times a day, and maintained this therapy (group A) and 53 patients who were switched from conventional GC treatment (15 on cortisone acetate and 38 on HC) to DR-HC (group B) administered orally in the morning in a fasting state, for a 48-month period. Out of the 40 patients excluded from the study, 30 patients did not meet the inclusion criteria, while 10 were lost to follow-up (Fig. 1). The switch to DR-HC was judged to be appropriate on clinical grounds in those patients who complained of fatigue and weakness, presented hyponatremia (<134 mmol/L) or hypoglycemia (≤2.78 mmol/L) or showed more than two comorbidities such as diabetes, osteoporosis/osteopenia, hypertension and central obesity. The switch from HC to DR-HC was made with an equivalent, while the dose was reduced from cortisone acetate to DR-HC taking into consideration the minor GC activity of cortisone acetate compared to HC and patients’ clinical characteristics.

All patients had disease duration of at least 5 years. Inclusion criteria were the following: age 18–75 years; diagnosis of AI; ongoing daily conventional GC treatment for at least 5 years. Exclusion criteria were pregnancy and lactation and AI secondary to adrenocortical carcinoma.

AI was diagnosed as recommended by international guidelines (13). Diagnosis of diabetes was made according to the ADA guidelines (14).

Overall, 44 patients had PAI, 31 with autoimmune polyglandular syndrome (APS) and 13 with isolated autoimmune AI (Table 1). Patients with celiac disease were on a stable gluten-free diet. Patients with PAI were also on stable treatment with fludrocortisone (0.05–0.1 mg/day, once). Among patients with APS and type 1 diabetes, four were in group A and six in group B.

**Table 1:** Distribution of patients with adrenal insufficiency.

Types of adrenal insufficiency	Cases (No. = 100)
Secondary adrenal insufficiency	
Hypocortisolism + hypothyroidism	12
Hypocortisolism + GH deficiency	11
Hypocortisolism + hypothyroidism + GH deficiency	10
Hypocortisolism + hypogonadism	8
Hypocortisolism + hypogonadism + hypothyroidism + GH deficiency	7
Hypocortisolism + hypogonadism + hypothyroidism	4
Isolated ACTH deficiency	4
Isolated primary adrenal insufficiency	11
Primary adrenal insufficiency in polyglandular autoimmune syndrome	
Addison’s disease + autoimmune thyroid disease	11
Addison’s disease + type 1 diabetes mellitus + autoimmune hypothyroidism	10
Addison’s disease + autoimmune hypothyroidism + celiac disease	10

The characteristics of patients with SAI and the other endocrine deficiency combinations are shown in Table 1. Patients with hypothyroidism were treated with levo-thyroxine at the average dose of 1 µg/kg. Patients with GHD were treated with somatotropin at the average dose of 0.4 mg/day. Males with hypogonadism were treated with an average dose of monthly injection of testosterone enanthate 250 mg. Premenopausal females were treated with a low dose of estrogen and progesterone therapy. No history of chronic GC use before substitutive treatment was known for patients with SAI.

All patients with SAI were on stable replacement treatment for the other deficiencies and maintained good and stable hormonal control during the whole follow-up. At baseline and after 48 months of DR-HC, respectively they had IGF-1 levels 137.5 ± 25.6 and 154.5 ± 29.6 ng/mL, FT4 levels 1.26 ± 0.36 and 1.19 ± 0.27 ng/dL, total testosterone 5.1 ± 1.6 and 5.7 ± 1.75 ng/dL (males) and total estrogens 104.2 ± 35.8 and 121 ± 28.2 pg/mL (females). Fourteen patients with SAI had type 2 diabetes (four in group A and ten in group B).

During the 48-month treatment period, the conventional GC and the DR-HC doses were changed based on the physician’s judgment of a patient’s need in both groups of patients (Table 2). Each patient received instructions for treatment in special or emergency situations. Patients treated with DR-HC were instructed to add a rescue dose of HC during an intercurrent illness or stress (5 or 10 mg according to severity of stress and symptoms).

**Table 2:** Dose adjustments according to the physician’s judgment during the 48 months of conventional glucocorticoid and dual-release hydrocortisone treatments.

Baseline dose	20 mg/day	25 mg/day	30 mg/day	35 mg/day	40 mg/day
Dose at 48 months of DR-HC					
20 mg/day (*n* = 32) 25 mg/day (*n* = 10) 30 mg/day (*n* = 4) 40 mg/day (*n* = 7)	30 0 0 0	1 8 0 0	0 1 2 0	0 1 0 0	1 0 2 7
**Baseline dose**	**12.5 mg/day**	**25 mg/day**	**37.5 mg/day**	**50 mg/day**	**62.5 mg/day**
Dose at 48 months of cortisone acetate					
12.5 mg/day (*n* = 1) 25 mg/day (*n* = 5) 37.5 mg/day (*n* = 8) 50 mg/day (*n* = 5) 62.5 mg/day (*n* = 1)	0 0 0 0 0	1 3 0 0 0	0 2 8 0 0	0 0 0 5 0	0 0 0 0 1
**Baseline dose**	**10 mg/day**	**15 mg/day**	**20 mg/day**	**25 mg/day**	**30 mg/day**
Dose at 48 months of hydrocortisone					
10 mg/day (*n* = 2) 15 mg/day (*n* = 12) 20 mg/day (*n* = 11) 25 mg/day (*n* = 1)	1 0 0 0	1 11 0 0	0 1 10 0	0 0 1 1	0 0 0 0
30 mg/day (*n* = 1)	0	0	0	0	1

The effects of the different doses of conventional GCs and DR-HC on metabolic parameters were also evaluated in all patients. However, only a minority of patients, 5/47 (11%) in group A and 8/53 (15%) in group B had their dose changed (Table 2) and no differences were found with the change in dosage (data not shown).

This study was carried out in accordance with the recommendations of the Paolo Giaccone Policlinico ethics committee with written informed consent from all subjects. All subjects gave written informed consent in accordance with the Declaration of Helsinki. The protocol was approved by the Paolo Giaccone Policlinico Ethics Committee.

### Study design

At baseline and after 48 months of conventional GCs and DR-HC treatment, clinical and metabolic parameters were extracted from our archive.

Anthropometric parameters such as BMI, systolic and diastolic blood pressure (SBP and DBP) and WC, measured at the midpoint between the lower rib and the iliac crest, were extracted. In addition, fasting lipids (total cholesterol (TC), HDL cholesterol (HDL-C), LDL cholesterol (LDL-C) and triglycerides (TG)), HbA1c and glycemia were obtained. The blood sample was performed about 2 h after GC administration (patients took the dose in the morning on waking) to avoid patients experiencing fatigue or other symptoms due to the delayed intake of the drug.

In patients with type 1 diabetes mellitus, the insulin requirement was calculated as total daily, basal and prandial insulin (U/day).

The FRS for estimating the 10-year risk for cardiovascular events was calculated on the basis of age, sex, total and HDL cholesterol, blood pressure and smoking status for 80 patients with normal glucose tolerance, while the UKPDS risk engine was calculated for 14 patients with type 2 diabetes mellitus. Six non-diabetic patients aged <30 years were excluded for estimation of FRS.

### Assays

Insulin, glycemia, HbA1c and lipids were measured with standard methods (Modular P800, Roche). LDL-C levels were calculated using the Friedewald formula (TC − (HDL + (TG/5))).

The conversion factors for the International System (SI) were as follows: glucose mg/dL vs mmol/L: 0.0555; TC and HDL-C mg/dL vs mmol/L: 0.0259; TG mg/dL vs mmol/L: 0.0113; HbA1c % vs mmol/mol: 10.93–23.5%.

### Statistical analysis

The Statistical Packages for Social Science SPSS version 19 (SPSS, Inc., IBM) were used for data analysis. The normality of quantitative variables was tested with the Shapiro–Wilk test. The baseline characteristics of the groups were presented as mean ± s.d. for continuous variables, while the rates and proportions were calculated for categorical data. The differences between the final and baseline data were evaluated with paired *t*-tests in a single group for quantitative variables and χ^2^ for categorical variables. The differences between the independent samples from groups A and B at 48 months were evaluated using the Student’s *t*-test. The differences between patients treated with high and low doses of GCs and between patients divided by disease etiology (PAI and SAI) are presented as mean ± s.d. A *P* value <0.05 was considered statistically significant.

## Results

The baseline characteristics of all patients with AI and subgroups (A and B) are shown in Table 3. During the 48-month period of observation 3 out of 47 patients had an adrenal crisis in group A (0.06%) and none in the group B.

**Table 3:** General characteristics of all patients and subgroups A and B at baseline.

	All	Group A	Group B	*P*^a^
	Baseline (No. = 100)	Baseline (No. = 47)	Baseline (No. = 53)	
	Subjects (%)	Subjects (%)	Subjects (%)	
Gender				
Male	32 (32%)	15 (31.9%)	17 (32.1%)	0.986
Female	68 (68%)	32 (68.1%)	36 (67.9%)	
Primary adrenal insufficiency (PAI)	44 (44%)	18 (38.3%)	26 (49.1%)	0.371
Secondary adrenal insufficiency (SAI)	56 (56%)	29 (61.7%)	27 (50.9%)	0.731
Arterial hypertension	29 (29%)	14 (29.8%)	15 (28.3%)	0.870
Osteoporosis/osteopenia	40 (40%)	10 (21.3%)	30 (56.6%)	<0.001
Visceral obesity	71 (74%)	25 (53.1%)	46 (86.7%)	0.125
Hypercholesterolemia	31 (31%)	8 (17%)	23 (43.4%)	0.073
Diabetes	24 (24%)	8 (17%)	16 (30.1%)	0.044
Replacement therapy at run-in				
Cortisone acetate	56 (56%)	20 (42.5%)	31 (67.9%)	0.082
Hydrocortisone	44 (44%)	27 (57.5%)	22 (32.1%)	0.160
	**Mean ± s.d.**	**Mean ± s.d.**	**Mean ± s.d.**	
Duration of disease (years)	14.7 ± 11.6	14.8 ± 11.5	14.5 ± 11.9	0.891
Age (years)	49.7 ± 22.1	49.9 ± 22.2	49.5 ± 21.9	0.874
Anthropometric parameters				
BMI (kg/m^2^)	26.6 ± 5.1	25.9 ± 5.3	27.1 ± 4.9	0.329
Waist circumference (cm)	95.5 ± 14	95 ± 14	96 ± 15	0.639
Systolic blood pressure (SBP) (mmHg)	113 ± 15	110 ± 14	116 ± 16	0.066
Diastolic blood pressure (DBP) (mmHg)	69 ± 9	68 ± 8	70 ± 9	0.286
Electrolytes				
Na (mmol/L)	138 ± 4	140 ± 2	137 ± 4	0.001
K (mmol/L)	4.7 ± 0.6	4.8 ± 0.4	4.5 ± 0.5	0.145
Metabolic parameters				
Total cholesterol (mmol/L)	5.22 ± 1.04	5.01 ± 0.75	5.30 ± 1.1	0.160
HDL cholesterol (mmol/L)	1.53 ± 0.48	1.61 ± 0.47	1.41 ± 0.52	0.250
Triglycerides (mmol/L)	1.50 ± 0.71	1.41 ± 0.76	1.56 ± 0.67	0.364
LDL cholesterol (mmol/L)	2.89 ± 0.92	2.64 ± 0.90	3.04 ± 0.91	0.062
Fasting glycaemia (mmol/L)	5.46 ± 3.13	5.40 ± 3.2	5.51 ± 3.12	0.886
HbA1c (mmol/mol)	41.3 ± 20.2	34.9 ± 21.6	47.4 ± 17	0.002

^a^*P* value between patients in group A and B at baseline.

At baseline, no differences between groups A and B were observed with regard to the etiology of AI, percentage of arterial hypertension, visceral obesity and hypercholesterolemia, duration of disease, anthropometric parameters, lipids and glycaemia (Table 3). However, patients in group B had a higher prevalence of osteoporosis/osteopenia (*P* < 0.001), diabetes (*P* = 0.044), lower sodium (*P* = 0.001) and higher HbA1c levels (*P* = 0.002), than group A (Table 4).

**Table 4:** Anthropometric and metabolic parameters in groups A and B at baseline and after 48 months of treatment.

	Group A (No. = 47)		*P*^a^	Group B (No. = 53)		*P*^b^	*P*^c^
	Baseline	48 months		Baseline	48 months		
	Mean ± s.d.	Mean ± s.d.		Mean ± s.d.	Mean ± s.d.		
Anthropometric parameters							
BMI (kg/m^2^)	25.9 ± 5.3	28 ± 5.47	<0.001	27.6 ± 4.96	26.6 ± 5.06	0.002	0.174
WC (cm)	94 ± 13.5	100 ± 12	0.001	98 ± 13	94 ± 12	0.015	0.020
Systolic blood pressure (mmHg)	111 ± 14	121 ± 14	0.001	118 ± 15	115 ± 16	0.461	0.037
Diastolic blood pressure (mmHg)	68 ± 8	70 ± 10	0.251	71 ± 9	66 ± 9	0.031	0.162
Metabolic parameters							
Total cholesterol (mmol/L)	5.01 ± 0.75	5.07 ± 0.71	0.136	5.45 ± 1.31	4.72 ± 0.72	0.006	0.006
HDL cholesterol (mmol/L)	1.56 ± 0.54	1.45 ± 0.33	0.137	1.52 ± 0.48	1.58 ± 0.39	0.284	0.015
Triglycerides (mmol/L)	1.41 ± 0.76	1.48 ± 0.72	0.131	1.60 ± 0.76	1.47 ± 0.83	0.393	0.751
LDL cholesterol (mmol/L)	2.64 ± 0.9	3.03 ± 0.76	0.018	3.08 ± 0.99	2.46 ± 0.69	0.005	<0.001
HbA1c (mmol/mol)	34.8 ± 22.8	43.1 ± 10.4	0.020	48.7 ± 16.9	39.1 ± 8.9	<0.001	0.028
Fasting glycemia (mmol/L)	5.45 ± 3.22	5.47 ± 2.57	0.960	5.65 ± 3.57	5.42 ± 3.51	0.549	0.225

^a^*P*: comparison between baseline and 48 months of treatment in group A; ^b^*P*: comparison between baseline and 48 months of treatment in group B; ^c^*P*: comparison between groups A and B at 48 months of treatment.

After 48 months of observation, patients in group A had a significant increase in BMI (*P* < 0.001), WC (*P* = 0.001), SBP (*P* = 0.001), LDL-C (*P* = 0.018) and HbA1c (*P* = 0.020) (Table 4). By contrast, patients in group B had a significant decrease in BMI (*P* = 0.002), WC (*P* = 0.015), DBP (*P* = 0.031), TC (*P* = 0.006), LDL-C (*P* = 0.005) and HbA1c (*P* < 0.001) (Table 4).

At 48 months of treatment lower values of WC (*P* = 0.020), SBP (*P* = 0.001), TC (*P* = 0.005), LDL-C (*P* < 0.001), HbA1c (*P* = 0.028) and higher values of HDL-C (*P* = 0.015) were observed in group B compared to group A (Table 4).

**Figure 2:**
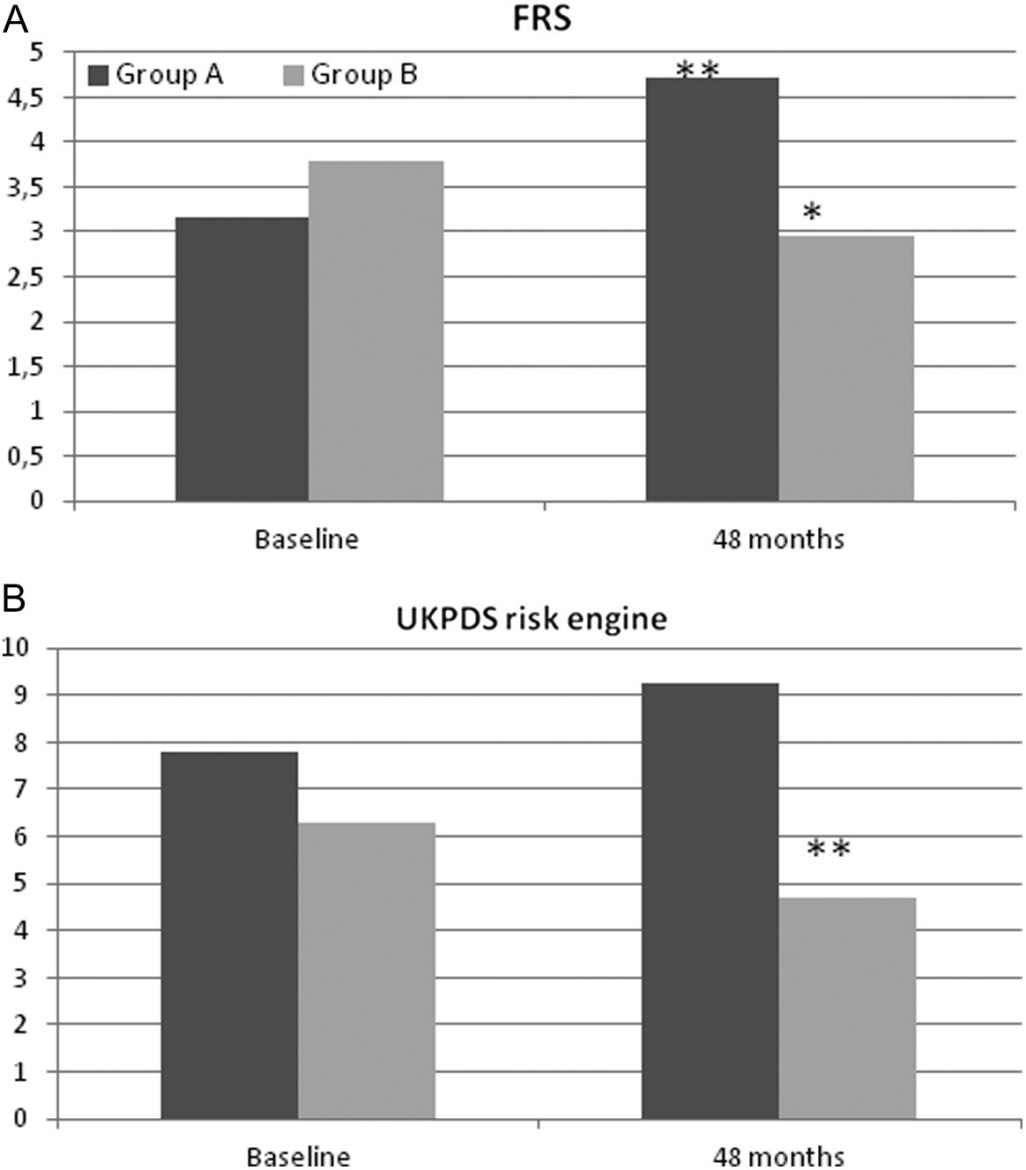
(A) Changes in FRS from baseline to 48 months for 74 non-diabetic patients of groups A and B. *P < 0.05, **P < 0.01; ***P < 0.001 vs baseline using the Student *t* test. Data are means (S.D.). (B) Changes in UKPDS risk engine from baseline to 48 months for 14 patients with type 2 diabetes mellitus in groups A and B. *P < 0.05, **P < 0.01; ***P < 0.001 vs baseline using the Student *t* test. Data are means (s.d.).

With regard to cardiovascular risk estimated by FRS, in non-diabetic patients a significant increase in group A (*P* = 0.002) and a significant decrease in group B (*P* = 0.015) was observed (Fig. 2A). On the other hand, for patients with type 2 diabetes mellitus, a significant decrease in the UKPDS risk engine (*P* = 0.002) was observed in patients in group B, while no change was shown in patients in group A (Fig. 2B).

No differences between patients switched from HC to DR-HC and patients switched from cortisone acetate to DR-HC were observed at baseline (data not shown).

The comparison between high and low doses of conventional GCs and DR-HC at 48 months of observation showed no significant differences for all parameters (Table 5).

**Table 5:** Anthropometric and metabolic parameters in patients treated with high and low doses of conventional GCs after 48 months of treatment.

	High doses of conventional GCs (No. = 16)	Low doses of conventional GCs (No. = 31)	*P*
	Mean ± s.d.	Mean ± s.d.	
Anthropometric parameters			
BMI (kg/m^2^)	29.6 ± 5.76	27.5 ± 5.21	0.264
WC (cm)	103.3 ± 13.6	99.5 ± 11.5	0.366
Systolic blood pressure (mmHg)	125 ± 15	119 ± 13	0.182
Diastolic blood pressure (mmHg)	71 ± 10	68 ± 9	0.377
Metabolic parameters			
Total cholesterol (mmol/L)	5.42 ± 0.66	5.19 ± 0.80	0.415
HDL cholesterol (mmol/L)	1.29 ± 0.34	1.37 ± 0.32	0.462
Triglycerides (mmol/L)	1.33 ± 0.44	1.61 ± 0.79	0.266
LDL cholesterol (mmol/L)	3.51 ± 0.74	3.05 ± 0.73	0.114
HbA1c (mmol/mol)	42.3 ± 9.34	44.8 ± 12.8	0.485
Fasting glycemia (mmol/L)	5.13 ± 0.91	5.74 ± 3.01	0.478
FRS	5.03 ± 4.39	4.26 ± 3.36	0.789

### Patients with diabetes

**Figure 3:**
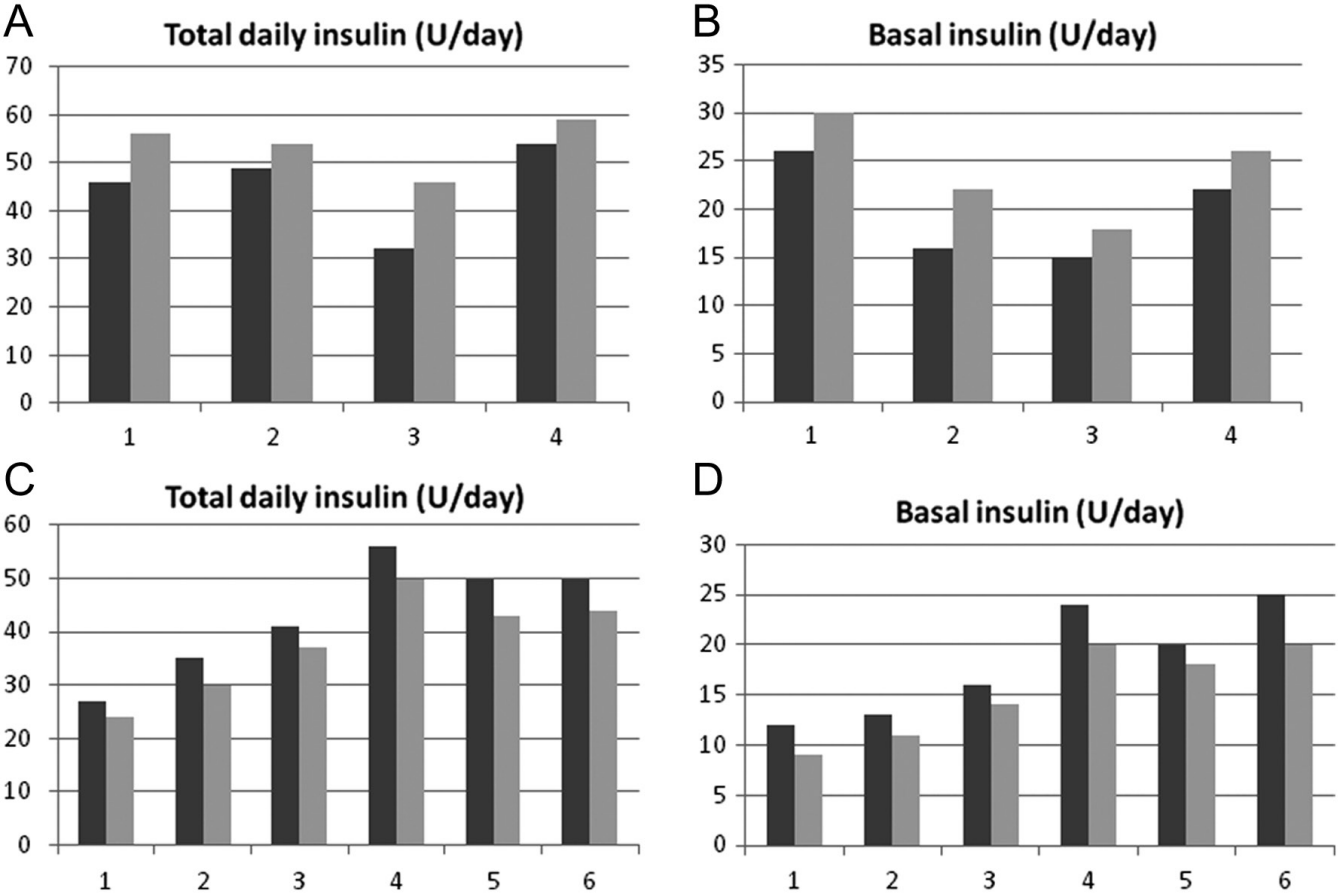
(A) Changes in total daily insulin (U/day) from baseline (dark gray line) to 48 months of treatment (light gray line) for four patients with type 1 diabetes mellitus in group A. (B) Changes in basal insulin (U/day) from baseline (dark gray line) to 48 months of treatment (light gray line) for four patients with type 1 diabetes mellitus in group A. (C) Changes in total daily insulin (U/day) from baseline (dark gray line) to 48 months of treatment (light gray line) for six patients with type 1 diabetes mellitus in group B. (D) Changes in basal insulin (U/day) from baseline (dark gray line) to 48 months of treatment (light gray line) for six patients with type 1 diabetes mellitus in group B. Data are single values for each patient.

In the total sample of patients analyzed, ten (four in group A and six in group B) had type 1 diabetes mellitus. After 48 months of treatment, an increase in total daily and basal insulin (U/day) was observed in group A (Fig. 3A and B). By contrast, a decrease in total daily and basal insulin (U/day) was observed in group B (Fig. 3C and D).

With regard to type 2 diabetes mellitus, after 48 months of observation, three new cases of type 2 diabetes occurred in group A, with a total of seven patients. In three cases in group A the antidiabetic treatment was changed (in two patients liraglutide was added to metformin and in one patient sitagliptin was added to metformin). In group B no new cases of diabetes occurred and no change in antidiabetic treatment was observed.

**Figure 4:**
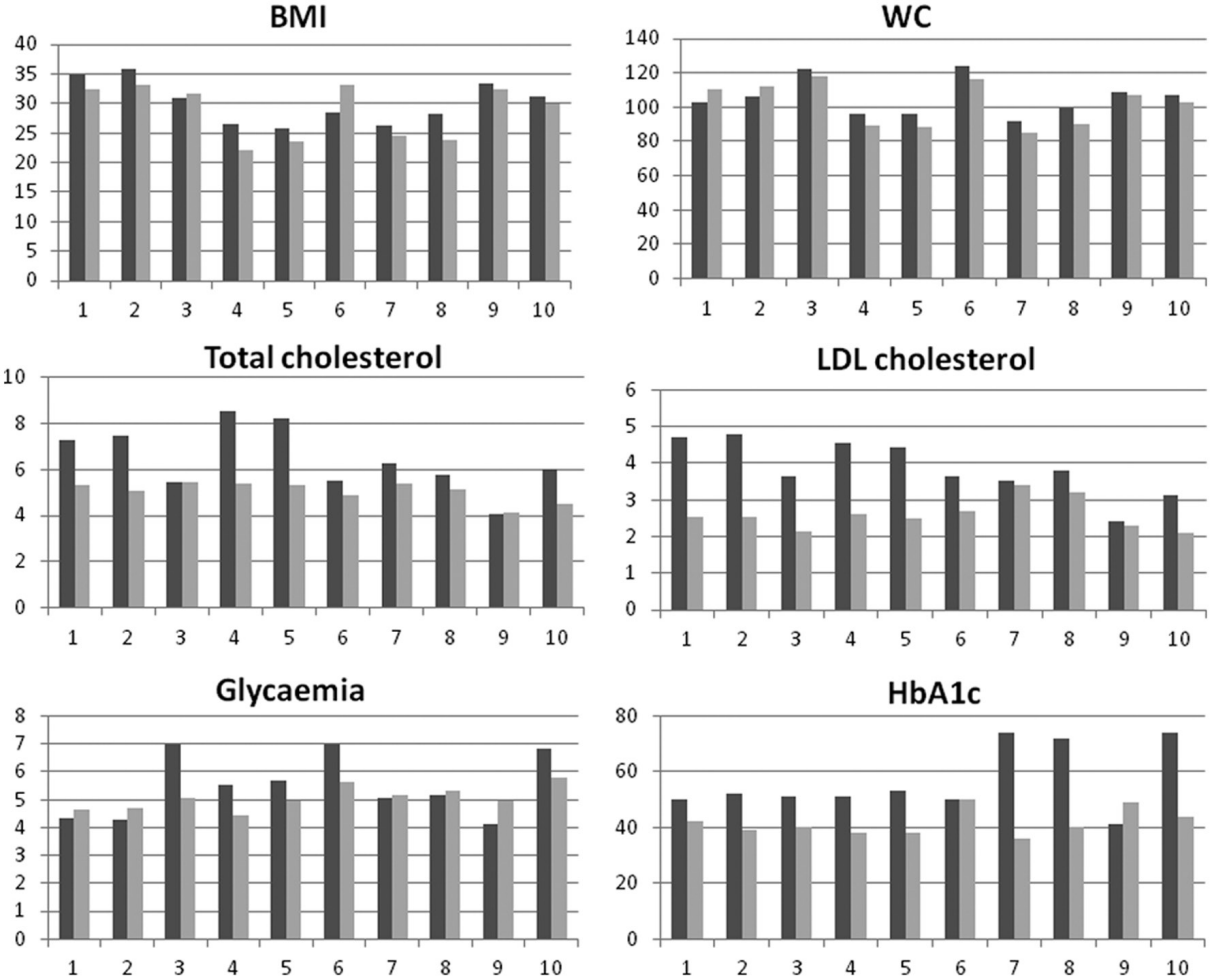
Changes in metabolic parameters from baseline (dark gray line) to 48 months of treatment (light gray line) for ten patients with type 2 diabetes mellitus in group B. BMI, body mass index; LDL, low density lipoprotein; WC, waist circumference. Data are single values for each patient.

In patients with type 2 diabetes mellitus in group B a decrease in BMI, WC, TC, LDL-C, fasting glycaemia and HbA1c was observed from baseline to 48 months of treatment (Fig. 4), while no differences were observed in patients in group A (data not shown).

### Patients lost to follow-up

Of the total of ten patients lost to follow-up, six were on conventional GC treatment and four were on DR-HC. One patient on conventional GC treatment was followed up for 12 months, 2 patients for 18 months and the other 3 patients for 24 months. Among patients treated with DR-HC, two patients were followed up for 6 months, one patient for 12 months and the other one for 18 months.

The reasons for loss of follow-up in the group of conventional GCs were moving to another city for five out of six patients, while one patient died. In the DR-HC group, two patients moved to another city, while the other two experienced severe fatigue, vomiting and hypotension. These two patients were treated with an additional dose of 10 mg of HC in the morning.

## Discussion

This study shows that in patients with AI the innovative therapy with DR-HC is associated with an improvement in anthropometric and metabolic parameters and in cardiovascular risk expressed through the FRS during a 48-month period, compared to conventional GCs, which are associated with a worsening of metabolic profile and cardiovascular risk.

Conventional GC therapy has been reported to be associated with increased mortality and morbidity in patients with hypoadrenalism compared to the general population, likely due to negative effects on metabolism (1, 15, 16, 17, 18). The effects of GCs on cardiovascular risk factors, such as obesity, hypertension, diabetes and dyslipidemia, are well known, as observed in hypercortisolism and in patients with AI on GC replacement therapy (19, 20, 21, 22). However, discordant results are reported about the different effects of low and high doses of conventional GCs. Some studies have reported a dose-dependent association between oral GCs and the risk of cardiovascular and cerebrovascular disease (23), mainly due to endothelial dysfunction (24, 25). By contrast, other studies do not show an association between either cardiovascular function or cardiovascular risk factors with reduced daily doses of HC (2, 26, 27).

In the current study, no differences in metabolic parameters and cardiovascular risk were observed between high and low doses either in patients treated with conventional GCs or in patients treated with DR-HC. However, a trend of increase in BMI, WC, SBP, TC and LDL-C was observed in patients treated with high doses of DR-HC and need to be further evaluated in a larger cohort of patients. Interestingly, patients treated with low doses of both standard GCs and DR-HC tended to have a progressive increase of the dose during the observation period.

A hypothesis of the GC impact on cardiovascular risk may be that GCs act both directly through ample expression of GC receptors in the cardiovascular system, including blood vessel walls and myocardium (28), and indirectly through deterioration of glucose and lipid metabolism (16, 29, 30).

The recent formulation of DR-HC has been shown to ensure an effective concentration of plasma cortisol, simulating the profile of physiological cortisol (31, 32). Several studies have shown that DR-HC treatment is associated with a reduction in weight, WC, systolic and diastolic blood pressure, HbA1c and lipids and an improvement of quality of life (6, 7, 9, 15). Furthermore, a recent study by our group documented a decrease in BMI, WC, HbA1c and an increase in HDL-C in patients treated with DR-HC for 36 months, showing a significant improvement in insulin secretion and sensitivity, even in patients with pre-diabetes (8).

In agreement with previous studies, in our study, we observed a significant worsening of BMI, WC and SBP, LDL cholesterol, HbA1c and FRS in patients treated with conventional GCs, while a significant improvement in DBP, BMI, WC, total and LDL cholesterol, HbA1c and FRS was observed in patients treated with DR-HC.

These findings are quite interesting because they show that cortisol-time exposure is strictly associated with a worsening of metabolism, likely influencing cardiovascular risk, even at low doses. Indeed, except for some patients (14 with cortisone acetate and 2 with hydrocortisone) treated with high doses of GCs, most of the patients on a conventional GC regimen were treated with low doses. In addition, in the group of patients treated with DR-HC, only one-third had high doses (35–40 mg/day). The findings of the current study suggest that the different profile of cortisol exposure, as in patients treated with DR-HC, may have an influence on metabolism and cardiovascular risk, more than the cumulative daily doses. Johansson *et al*. reported that switching from immediate-release hydrocortisone to DR-HC using the same total daily dose leads to a 22% reduction in cortisol exposure, as estimated by the area under the curve (31). Therefore, the beneficial effects of DR-HC might be explained by a reduction in cortisol exposure, meaning more physiological circadian fluctuations of cortisol concentrations, from a relatively high and possibly immunosuppressive dose of conventional GCs to that equivalent to a standard replacement dose. Similarly, the large weight gain and consequent metabolic derangement in the group of patients treated with conventional GCs may be the effect of a prolonged exposure to supraphysiological cortisol levels.

The improvement in FRS in patients switched from conventional treatment to DR-HC is an interesting and innovative finding that may be explained by a reduction of GC exposure, notably overnight, and may be mediated by direct and indirect effects (improvement in anthropometric parameters, lipids and glucose metabolism).

In line with a previous study (7), we also found a decrease in insulin requirement in patients with type 1 diabetes mellitus treated with DR-HC, compared to an increase in those patients maintaining conventional GC therapy. Conventional GC therapy has already been reported negatively to affect insulin requirement in patients with APS and type 1 diabetes, compared to patients with only type 1 diabetes, mainly due to non-physiological daily GC availability, notably in the evening, inducing an increase in insulin resistance (33, 34).

The improvement in BMI, WC, fasting glycemia, HbA1c, TC and LDL-C in patients with type 2 diabetes treated with DR-HC partially confirms the results of previous studies. Indeed, Giordano *et al*. showed an improvement in HbA1c, but not in fasting glycaemia in diabetic patients treated with DR-HC (7). By contrast, Nilsson *et al*. did not show any differences in glucose control and lipid profile, except for an improvement in HDL-C, after a switch from conventional GCs to DR-HC (15). On the other hand, in our study, the maintenance of conventional GCs did not affect metabolism and anthropometric parameters, probably due to the addition of antidiabetic treatment in three out of four patients with type 2 diabetes mellitus.

The main limitations of the current study are the type of study, which is a real-world evidence-based one, and the small number of patients enrolled. Due to the rarity of the disease, a non-randomized design was chosen to evaluate as many patients as possible in the shortest possible time. However, this design introduces a selection bias and does not make it possible to control exposure or outcome assessment. Another limitation is that lifestyle, dietary habits, alcohol consumption, exercise and socioeconomic status were not verified. Although they are advised to increase the dose, we cannot be certain that patients will be compliant during the follow-up, due to the fear they frequently have of weight gain related to GC therapy. Lastly, the cohort of patients was quite heterogeneous (patients with PAI and SAI) and was characterized by different metabolic alterations, due to other hormonal deficiencies. Indeed in SAI, other pituitary deficiencies or replacement therapies, such as growth hormone, are to be considered as an additional risk factor for metabolic disorders.

However, all patients enrolled in the study were on stable replacement treatment and maintained good and stable hormonal control during the whole follow-up, thus not affecting, in our opinion, the outcomes of the study.

Despite these limitations, the strength of the study is represented by the long duration, the evaluation of cardiovascular risk and the inclusion of patients switched from cortisone acetate to DR-HC, which has not been widely described before.

In conclusion, our preliminary data, extracted from real-life clinical practice, suggest that 48-month treatment with DR-HC is associated with an improvement in anthropometric and metabolic parameters and cardiovascular risk, evaluated by FRS and the UKPDS risk engine, compared to conventional GC therapy, regardless of the dose used. However, further prospective studies performed in a larger cohort of patients are required in order to verify our data.

## Declaration of interest

The authors declare that there is no conflict of interest that could be perceived as prejudicing the impartiality of the research reported.

## Funding

This research did not receive any specific grant from any funding agency in the public, commercial or not-for-profit sector.

## Author contribution statement

V G, C D, A S, A L C, A N C and C G had full control of the study design, data analysis and interpretation and preparation of the article. All authors were involved in planning the analysis and drafting the article. The final draft article was approved by all the authors.
